# Apocynin Improves Insulin Resistance through Suppressing Inflammation in High-Fat Diet-Induced Obese Mice

**DOI:** 10.1155/2010/858735

**Published:** 2011-02-21

**Authors:** Ran Meng, Da-Long Zhu, Yan Bi, Dong-Hui Yang, Ya-Ping Wang

**Affiliations:** ^1^Department of Endocrinology, Nanjing Drum Tower Hospital, Nanjing University School of Medicine, 321 Zhongshan Road, Nanjing 210008, China; ^2^Department of Medical Genetics, Nanjing University School of Medicine, 22 Hankou Road, Nanjing 210093, China; ^3^Jiangsu Key Laboratory of Molecular Medicine, Nanjing University, 22 Hankou Road, Nanjing 210093, China

## Abstract

We investigated the effects of apocynin on high-fat diet- (HFD-) induced insulin resistance in C57BL/6 mice. After 12 weeks of HFD, the mice that exhibited insulin resistance then received 5 weeks of apocynin (2.4 g/L, in water). Following apocynin treatment, fasting glucose, insulin, and glucose tolerance test showed significant improvement in insulin sensitivity in HFD-fed mice. We demonstrated that serum levels of tumor necrosis factor-*α* (TNF-*α*), interleukin-6 (IL-6), and leptin were remarkably reduced with apocynin treatment. We also found that mRNA expression of TNF-*α*, IL-6, and monocyte chemoattractant protein-1 (MCP-1) in the liver and mRNA expression of TNF-*α*, IL-6, MCP-1, and leptin in adipose tissue were suppressed by apocynin. Furthermore, the activity of transcription factor NF-*κ*B in the liver was significantly suppressed with apocynin treatment. These results suggest that apocynin may reduce inflammatory factors in the blood, liver, and adipose tissue, resulting in amelioration of insulin resistance in HFD-fed mice.

## 1. Introduction

Insulin resistance is one of the critical mechanisms of the type 2 diabetes [1]. Increasing evidence suggests that inflammation plays an important role in the development of insulin resistance [2]. The inflammatory status of the liver and adipose tissue, the main insulin-resistance-related organs, is critical for insulin sensitivity of the whole body [3, 4].

During development of inflammation in the liver, hepatocytes are exposed to the increasing cytokines such as tumor necrosis factor-*α* (TNF-*α*), interleukin-6 (IL-6), and monocyte chemoattractant protein-1 (MCP-1) [5]. These cytokines were regulated by nuclear factor kappa-B (NF-*κ*B) [6, 7]. A number of studies demonstrated that hepatic NF-*κ*B activation was linked to the development of the whole body inflammation, resulting in insulin resistance [8, 9]. Additionally, adipose tissue is an active endocrine organ that secretes numerous inflammation-related adipokines, cytokines, and chemokines. The expression of TNF-*α*, IL-6, MCP-1, and leptin is upregulated in the adipose tissue of obese mice which is closely associated with insulin resistance [10–12]. 

Interestingly, some studies reported that oxidative stress could induce inflammation during development of insulin resistance [13, 14]. Oxidative stress which is induced by excess reactive oxygen species (ROS) could activate NF-*κ*B [15]. Antioxidants have been reported to potently suppress the activation of NF-*κ*B in the liver [16–18]. Furthermore, increased oxidative stress in adipocytes causes dysregulation of cytokines and adipokines, including adiponectin, MCP-1, IL-6, and plasminogen activator inhibitor-1 (PAI-1) [19, 20]. Accordingly, antioxidants have been also reported to decrease inflammation in adipose tissue and ultimately ameliorate insulin resistance [21, 22].

Apocynin (4-hydroxy-3-methoxy-acetophenone) is a constituent of the Himalayan herb Picrorhiza kurrooa Royle (Scrophulariaceae) which is regarded as an inhibitor of nicotinamide adenine dinucleotide phosphate (NADPH)-oxidase and is widely used as an antioxidant in research. In addition, apocynin also exhibits anti-inflammatory effects in previous studies [23, 24]. It was demonstrated that apocynin suppressed NF-*κ*B in spontaneously hypertensive rats [25], downregulated TNF-*α* protein expression in coronary arteries [26], decreased vascular cell adhesion molecule-1 (VCAM-1) induced by TNF-*α* in vein endothelial cells [27], reduced polymorphonuclear granulocyte chemotaxis [28], inhibited peroxynitrite in airway lumen in mice [29], attenuated inflammation-mediated cartilage destruction activity in human articular cartilage [30], and reduced cyclooxygenase (COX)-2 expression in human monocytes [31]. Since the critical role of inflammation in genesis of insulin resistance, it is reasonable to speculate that apocynin could improve insulin resistance through suppressing inflammation. However, no studies were conducted to examine the effects of apocynin on inflammation and its subsequent effects on insulin resistance.

In this study, C57BL/6 mice were fed with high-fat diet (HFD) to establish insulin resistance for investigating the effects of apocynin on the expression of inflammatory factors in blood, liver, and adipose tissue, and then evaluating the improvement of insulin sensitivity with apocynin treatment.

## 2. Methods

### 2.1. Animals and Experimental Procedures

Four-week-old male C57BL/6 mice used in this study were purchased from animal center, Yangzhou University (Yangzhou, Jiangsu, China). The mice were fed either with high-fat diet (60% kcal fat, 20% kcal carbohydrates, 20% kcal protein, Guangzhou animal experiment center, Guangzhou, China), or with normal chow diet (10% kcal fat, 70% kcal carbohydrates, 20% kcal protein) after one week's acclimation period. Mice were kept on a 12-hour light, 12-hour dark cycle and fed *ad libitum*. After 12 weeks of feeding, the HFD-fed mice showed obvious phenotypes of insulin resistance compared to the normal diet control group (ND-Con). We then randomly divided them into two groups (6 mice per group). One group received normal water (HFD control group, HFD-Con), while the other group received apocynin (2.4 g/L) dissolved in drink water (apocynin group, HFD-Apo) for another five weeks with HFD. Mice were sacrificed after fasting for 12 hours. After given ketamine-xylazine (130/8.8 mg/kg), their tissues were harvested, weighed, snap frozen in liquid nitrogen, and stored at −80°C until use. Dietary intake and body weight were recorded every week. All procedures were approved by the Nanjing University's Animal Care and Use Committee.

### 2.2. Plasma Parameters

Mice were fasted for 12 hours, and blood samples were collected from the orbital sinus under anesthesia. After centrifugation at 15,000× g for 1 min, the supernatants of the blood samples were separated and subjected to measurements of leptin, TNF-*α*, and IL-6 levels with the ELISA kits (RayBiotech, Norcross, GA, USA). Plasma insulin was determined with Mouse Insulin ELISA Assay kit (Linco, Charles, MO, USA).

### 2.3. Intraperitoneal Glucose Tolerance Tests (IPGTTs)

Mice were fasted for 6 hours prior to IPGTTs. Glucose was injected intraperitoneally with the concentration of 1 g/kg body weight. Blood glucose measurement were obtained from the tail at 0, 30, 60, and 120 min. Blood glucose levels were determined at indicated intervals with One Touch Profile Glucose Meter (Johnson & Johnson, New Brunswick, NJ, USA).

### 2.4. Quantitative PCR Analysis

Total RNA was extracted from the liver and epididymal adipose tissue of mice by Trizol (Takara Bio, Shiga, Japan). Synthesis of cDNA was performed by M-MLV reverse transcriptase (Toyobo, Osaka, Japan). Quantitative real-time PCR was performed with SYBR Premix Ex Taq (Takara Bio, Shiga, Japan), using the StepOne Real-Time PCR system according to the manufacturer's instructions (Applied Biosystems, Foster City, CA, USA). The primer pairs used in the present study are listed in Table 1. Final results were normalized against *β*-actin gene expression. 

### 2.5. NF-*κ*B Activity Assay

Nuclear protein extracts were prepared from liver tissue with the nuclear extract kit (Active Motif, Carlsbad, CA) according to the manufacturer's instructions. Protein concentrations were then quantified with a Bradford assay, and equal amounts of protein were used in a colorimetric NF-*κ*B assay specific for the activated form of p65 subunit of NF-*κ*B (Trans AM NF-*κ*B p65 kit; Active Motif, Carlsbad, CA).

### 2.6. Statistical Analysis

Data are expressed as the means ± SEM. *P* values were determined by one-way ANOVA followed by LSD and differences were considered significant if *P* < .05.

## 3. Results

### 3.1. Effect of Apocynin on Growth Parameters

**Figure 1:**
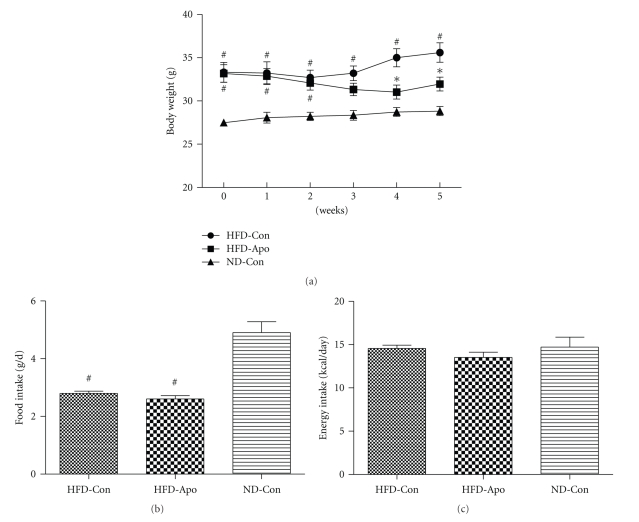
Effect of apocynin on body weight, food intake, and energy intake. C57BL/6 mice were fed with HFD for 12 weeks to induce insulin resistance. After 12 weeks of feeding, the HFD-fed mice were divided into two groups. The HFD-fed mice in one group were treated with apocynin (2.4 g/L) in drink water (HFD-Apo, *n* = 6) for another 5 weeks HFD feeding; the HFD-fed mice in the other group were employed as HFD controls (HFD-Con, *n* = 6). Normal control mice were fed a normal diet and untreated with apocynin (ND-Con, *n* = 6). (a) Body weight of three groups. The body weight of the HFD-Apo group was lower than that of the HFD-Con group since the fourth week after apocynin treatment. (b) Food intake. The food intake of the HFD-Apo group and HFD-Con group were lower than that in the ND-Con group. (c) Energy intake. There are no differences among groups. ^#^ *P* < .05  versus ND-Con group, *P < .05 versus HFD-Con group.

The effect of apocynin on body weight in HFD-fed mice was shown in Figure 1(a). The body weight of HFD-Apo group and HFD-Con group were similar (*P* > .05) and significantly higher than that in the ND-Con group until 3 weeks after the treatment of apocynin (*P* < .05). From the fourth week to the end of the experiment, the body weight of HFD-Apo group was clearly lower than that of HFD-Con group (*P* < .05). The mean dietary intake during the experiment was significantly lower in the HFD-Con and HFD-Apo groups than that in the ND-Con group (*P* < .05, Figure 1(b)), while energy intake has no significant difference among groups (*P* > .05, Figure 1(c)).

### 3.2. Effect of Apocynin on Insulin Sensitivity, Fasting Glucose, and Fasting Insulin

**Figure 2:**
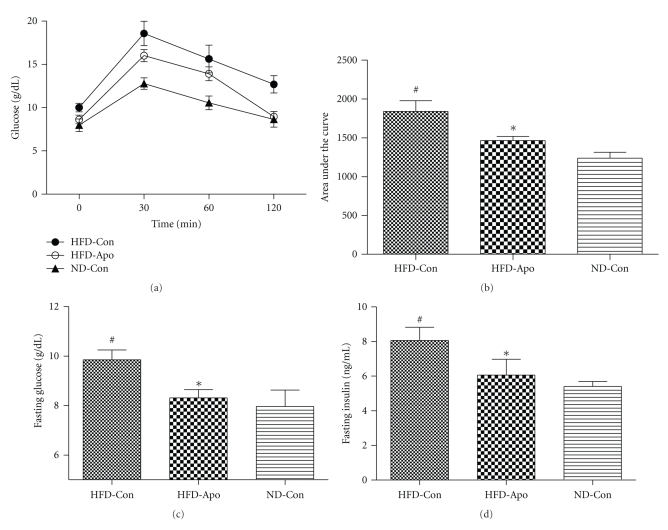
Effect of apocynin on obesity-induced glucose intolerance. (a) IPGTTs was performed by injection intraperitoneally of 1 g/kg glucose after 6-hour fasting, and blood samples were drawn from tail veins after 0, 30, 60, and 120 min. (b) Area under the curve was measured as a parameter of insulin resistance. Significant difference was detected between HFD-Apo group and HFD-Con group. (c) Fasting glucose levels. There is a significant difference between HFD-Apo group and HFD-Con group. (d) Fasting insulin levels. The fasting insulin level of the HFD-Apo group is lower than that of HFD-Con group. ^#^ *P* < .05 versus ND-Con group, *P < .05 versus HFD-Con group.

IPGTTs were performed before the sacrifice of the mice to assess glucose tolerance (Figure 2(a)). Area under the curve (AUC) parameter was employed as the index of glucose tolerance (Figure 2(b)). AUC was significantly higher in the HFD-Con group compared to the ND-Con group (*P* < .05). The HFD-Apo group had remarkably lower AUC than the HFD-Con group (*P* < .05). At the end of the IPGTTs, fasting glucose and fasting insulin levels were significantly higher in HFD-Con group than ND-Con group (*P* < .05). Apocynin administration significantly improved fasting glucose (Figure 2(c)) and fasting insulin in HFD-Apo group compared to the HFD-Con group (Figure 2(d)) (*P* < .05, resp.)

### 3.3. Effects of Apocynin on Systemic Inflammation

**Figure 3:**
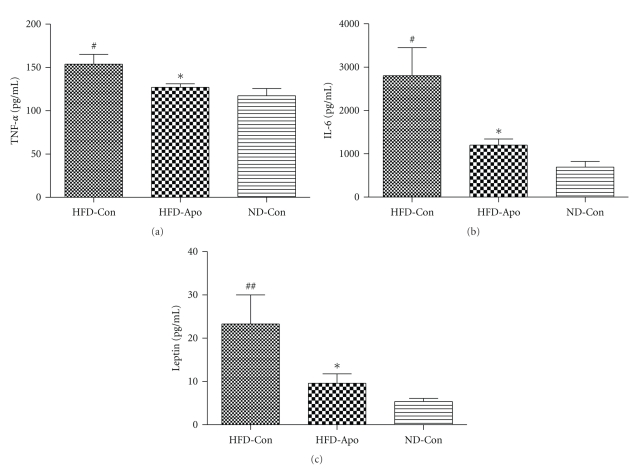
Effects of apocynin on the plasma inflammatory markers. (a) Plasma TNF-*α* levels. It was shown that apocynin decreased the level of plasma TNF-*α* in HFD-Apo group compared to the HFD-Con group. (b) Plasma IL-6 levels. The plasma IL-6 level in HFD-Apo group is lower than that in HFD-Con group. (c) Plasma leptin levels. Apocynin decreased the level of plasma leptin in HFD-Apo compared with the HFD-Con group. ^#^ *P* < .05 versus ND-Con group, ^##^ *P* < .01 versus ND-Con group, *P < .05 versus HFD-Con group.

Serum inflammatory cytokines levels are shown in Figures 3(a)–3(c). The levels of TNF-*α*, IL-6, and leptin of HFD-Con group were remarkably higher than ND-Con group (*P* < .05, resp.). Apocynin treatment significantly decreased the levels of TNF-*α*, IL-6, and leptin in the serum (*P* < .05, resp.). We then further tested inflammation related gene expression in adipose tissue and liver.

### 3.4. Effects of Apocynin on Inflammation-Related Gene Expression in Adipose Tissue

**Figure 4:**
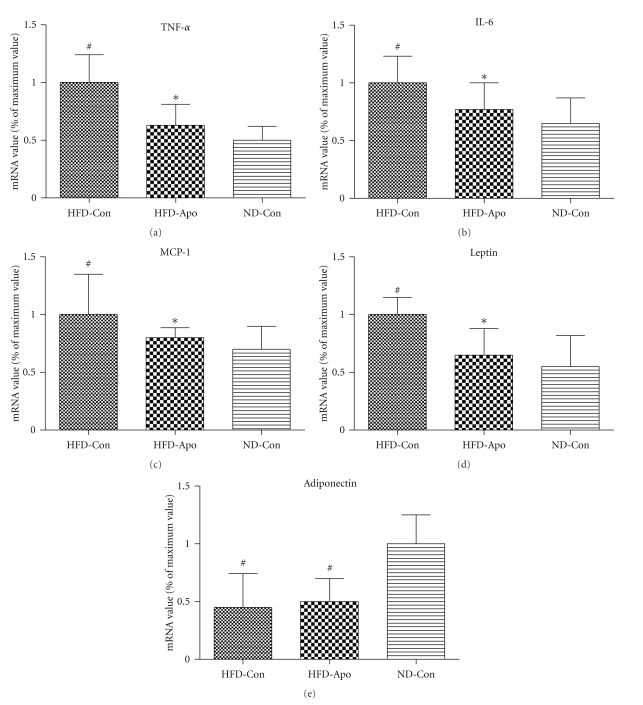
Effects of apocynin on inflammation-related genes expression in adipose tissue. The levels of mRNA expression of TNF-*α*, IL-6, MCP-1, leptin, and adiponectin in the epididymal adipose tissue were determined by quantitative PCR. (a) TNF-*α*, (b) IL-6, (c) MCP-1, (d) Leptin, and (e) Adiponectin. Apocynin decreased the gene expression of TNF-*α*, IL-6, MCP-1, and leptin in HFD-Apo compared to the HFD-Con group. The gene expression of adiponectin did not change among three groups. ^#^ *P* < .05 versus ND-Con group, *P < .05 versus HFD-Con group.

In adipose tissue, the HFD-Con group exhibited significantly higher gene expression of TNF-*α*, IL-6, MCP-1, and leptin and lower expression of adiponectin than ND-Con group (*P* < .05, resp.). Apocynin treatment obviously decreased TNF-*α*, IL-6, MCP-1, and leptin expression (*P* < .05, resp.) (Figures 4(a)–4(d)). However, there was no change in expression of adiponectin after treatment with apocynin (*P* > .05) (Figure 4(e)).

### 3.5. Effects of Apocynin on Inflammation-Related Gene Expression and NF-*κ*B Activity in the Liver

**Figure 5:**
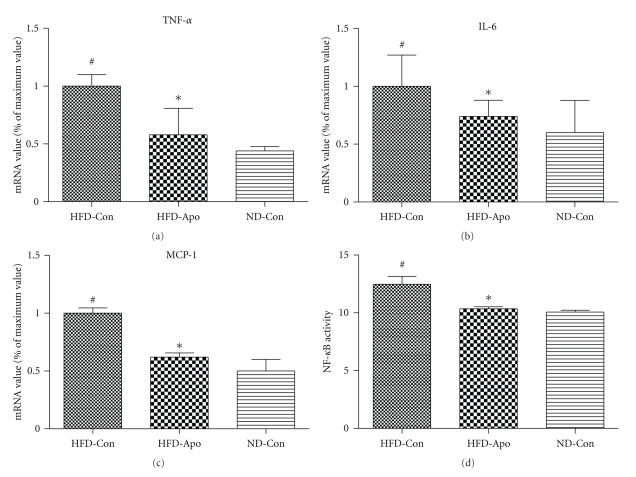
Effects of apocynin on inflammation-related genes expression and the activity of NF-*κ*B in the liver. Apocynin treatment decreased the gene expression of (a) TNF-*α*, (b) IL-6, (c) MCP-1 in HFD-Apo group compared to HFD-Con group. (d) Hepatic NF-**κ**B activity was tested by the special kit (Trans-AM NF-**κ**B kit). The hepatic NF-**κ**B activity is lower in the HFD-Apo group than that in the HFD-Con group.

In the liver, gene expression TNF-*α*, IL-6, and MCP-1 were significantly lower in the HFD-Apo than the HFD-Con group (*P* < .05, resp.) (Figures 5(a)–5(c)). We further tested NF-*κ*B activity in liver nuclear extract samples from the mice of three experiment groups. We found that significantly less NF-*κ*B activity was derived from apocynin-treated mice than those derived from untreated HFD-fed mice (*P* < .05) (Figure 5(d)).

## 4. Discussion

Our present study indicates that apocynin administration in HFD-fed C57BL/6 mice can improve insulin sensitivity, reduce the diverse plasma inflammatory cytokines, suppress gene expression of inflammation-related molecules in both liver and adipose tissue, and decrease the activity of transcription factor NF-*κ*B in liver tissue. These results suggest that apocynin could ameliorate insulin resistance through the suppression of inflammation in HFD-fed C57BL/6 mice.

To reflect modern dietary conditions, we employed C57BL/6 mice fed with HFD (60% kcal from fat) for 12 weeks which is a simple insulin resistance model. HFD-fed mice exhibited higher body weight than normal diet mice. Although food and energy intakes were not significantly different, the body weight of the HFD-Con group was increased, whereas that of the HFD-Apo group was decreased and close to ND-Con group. The HFD-fed mice exhibited significantly higher fasting glucose, fasting insulin levels, and AUC of IPGTT than normal control mice. Apocynin treatment dramatically decreased these indexes of insulin resistance.

Apocynin is an antioxidant which can be used as an inhibitor of NADPH oxidase. Recently, there have been a few studies that implicated that apocynin may improve insulin resistance which are consistent with the results of the present study. Winiarska and colleagues found that apocynin could inhibit renal gluconeogenesis action by suppressing oxidative stress, resulting in hypoglycaemic response in diabetic rabbits [32]. Another study indicated that apocynin administration decreased lipid peroxidation and hydrogen peroxide in adipose tissue which led to the improvement of plasma glucose and insulin resistance in KKAy mice, a transgenic animal model of type 2 diabetes [33]. Also, the antioxidative effect of apocynin was protective in diabetic nephropathy [34, 35], in diabetic endothelial dysfunction [36, 37], and in diabetic mitochondrial dysfunction in skeletal muscle [38]. However, none of the previous studies focused on the anti-inflammatory effect of apocynin on insulin resistance. 

Inflammation has been defined as an important contributor to insulin resistance in last century. Overproduction of inflammatory factors can cause insulin resistance. In contrast, suppressing inflammatory factors may ameliorate insulin resistance [39, 40]. The molecular pathways that link inflammation and insulin resistance include a variety of cytokines and adipocytokines such as TNF-*α*, IL-6, MCP-1, leptin, and adiponectin [2]. High circulating levels of TNF-*α*, IL-6, and leptin are recognized as markers of insulin resistance [10, 41, 42].

The liver and adipose tissue are two major insulin action organs. The inflammatory status of the liver and adipose tissue could directly impact the systemic inflammatory status that accompanies insulin resistance. Inflammation-related transcription factor NF-*κ*B in the liver is regarded as an important factor for inducing insulin resistance. Increased hepatic NF-*κ*B activity could cause profound hepatic insulin resistance and moderate systemic insulin resistance [8]. Furthermore, as a transcription factor, NF-*κ*B regulates expression of many kinds of cytokines in the liver. Once NF-*κ*B is activated, the gene expression of TNF-*α*, IL-6, and MCP-1 is inordinately increased [6, 7]. The endocrine function of adipose tissue and the association of obesity result in chronic low-grade inflammation and reinforce the concept of the central role of adipose tissue in mediating obesity-linked insulin resistance [43]. It has been discovered that the adipose tissue in fact contributes 15–35% of the body's basal circulating IL-6 and a large rate of whole body TNF-*α* [44]. Adipose tissue macrophages (ATMs) are proved to be predominantly responsible for the elevated production of TNF-*α* [45] and IL-6 during obesity [46]. MCP-1 could contribute macrophage infiltration into adipose tissue and affect the number of ATMs in adipose tissue, which in turn impacts the expression of TNF-*α* and IL-6. Moreover, leptin is almost exclusively expressed in adipose tissue. So gene expression of leptin in adipose tissue directly affects leptin level in the blood. 

Apocynin is a traditional antioxidant. However, Hart and his colleagues found that apocynin could reduce inflammation as well as superoxide anion [47]. Afterwards, a number of studies have demonstrated that apocynin could modulate the production of inflammatory factors production both *in vitro* [27–31] and* in vivo* [25, 33, 48]. In the present study, we investigated the effect of apocynin on the inflammatory status through HFD-induced insulin resistance in C57BL/6 mice. Our results show that the gene expression of TNF-*α*, IL-6, and MCP-1 in the liver and the gene expression of TNF-*α*, IL-6, MCP-1, and leptin in adipose tissue were upregulated after HFD feeding in mice. Apocynin administration suppressed the expression of these inflammatory agents, resulting in the reduction of circulating TNF-*α*, IL-6, and leptin levels that normally accompany insulin resistance. 

Furthermore, we found that apocynin could reduce the activity of hepatic NF-**κ**B which contributes to the improvement of insulin resistance both in the liver and whole body. How can the antioxidant apocynin decrease the NF-*κ*B activity? There is compelling evidence that in many cell types, ROS such as hydrogen peroxide and superoxide anion have a major effect on the release and nuclear translocation of the NF-*κ*B complex and on the induction of NF-*κ*B target genes [49, 50]. NADPH oxidase, a major source of ROS, triggers the production of superoxide anion [51, 52]. Hence, it is reasonable that apocynin, an inhibitor of NADPH oxidase, suppresses NF-*κ*B activity. Our results are in agreement with a study conducted by Pechanova and his colleagues who used apocynin in spontaneously hypertensive rats. After six weeks of treatment, apocynin significantly decreased the activity of NF-*κ*B in the left ventricle [25]. Although in the present study, we have proved that apocynin could ameliorate inflammatory status and suppress the activity of NF-*κ*B in the liver, changes in other inflammatory signaling pathways and insulin signaling remain unknown. Further investigations will be conducted in our lab.

In conclusion, apocynin has the potential to improve insulin sensitivity through ameliorating inflammatory status in the blood, liver, and adipose tissue of the C57BL/6 mice in which insulin resistance was induced by HFD. Hence, apocynin is a promising agent for treating insulin resistance.

## Figures and Tables

**Table 1:** Primer pairs used in present study.

Gene	Forward primer	Reverse primer
TNF-*α*	CAGGAGGGAGAACAGAAACTCCA	CCTGGTTGGCTGCTTGCTT
IL-6	TCCAGTTGCCTTCTTGGGAC	GTGTAATTAAGCCTCCGACTTG
MCP-1	CAATCAATGCCC CAGTCAC	GATTCTTGGGTTGTGGGAGTG
Leptin	GATGACACCAAAACCCTCATC	GCCACCACCTCTG TGGAGTAG
Adiponectin	GTCAGTGGATCTGACGACACCAA	ATGCCTGCCATCCAACCTG
Actin	CATCCGTAAAGACCTCTATGCCAAC	ATGGAGCCACCGATCCACA
